# Progressive dementia with seizures in an HIV infected lady

**DOI:** 10.11604/pamj.2017.27.242.9391

**Published:** 2017-08-02

**Authors:** Joe James, James Jose

**Affiliations:** 1 Department of Internal Medicine, Government Medical College Kozhikode, Kerala, India; 2 Department of Neurology, Government Medical College Kozhikode, Kerala, India

**Keywords:** HIV dementia, HIV, Magnetic Resonance Imaging, computed tomography

## Image in medicine

A 40-year-old female presented with progressive memory loss over the past 1-month. She had one episode of generalized tonic clonic seizure 2-months back, but did not take any treatment. She had significant involuntary weight loss. On examination she was emaciated and had a BMI of 16.4. Neurological examination showed cognitive decline in the form of loss of recent memory, judgment and abstract thinking, with preserved language and visuospatial orientation. There were no focal neurological deficits. She was tested positive for HIV. Her CD4+ count was 21/μL. Computed tomography of the brain revealed bilateral hypodensities in the periventricular area (A). MRI of the brain showed bilateral symmetrical periventricular white matter FLAIR hyperintensities in frontal and parietal lobes with diffuse brain atrophy suggestive of HIV encephalopathy (B). She was started on combination antiretroviral therapy with tenofovir, lamivudine and efavirenz and discharged. HIV encephalopathy refers to neurocognitive disorders associated with HIV infection. It usually occurs in late stages of HIV-infection and correlates with advanced immunosuppression. Earliest recognizable findings include impairment of attention and concentration. This progresses to frank dementia, personality changes and motor abnormalities. Neuroimaging typically shows global cerebral atrophy with symmetrical white matter hyperintensities predominantly in the periventricular area. Lesions of progressive multifocal leukoencephalopathy can mimic HIV encephalopathy, but are usually asymmetric, involve the peripheral “U” fibres first and have predilection for parieto-occipital lobes. Treatment of HIV encephalopathy is initiation or intensification of anti-retroviral therapy, and psychomotor improvement is usually seen after therapy.

**Figure 1:**
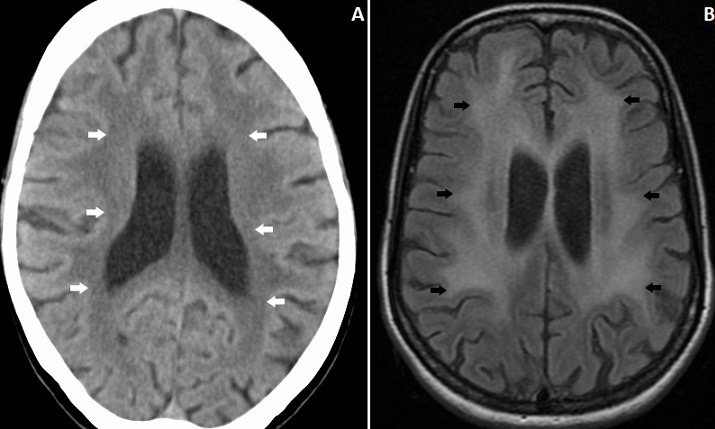
A) axial CT showing periventricular hypodensity (arrows); B) MRI Brain FLAIR image highlighting the periventricular lesions

